# Supplementary material for: Giardia intestinalis reshapes mucosal immunity toward a Type 2 response that attenuates inflammatory bowel-like diseases
Source: bioRxiv. 2024 Mar 6:2024.03.02.583119. Preprint. [Version 1] doi: 10.1101/2024.03.02.583119 (PMC11188066; doi:10.1101/2024.03.02.583119)
Supplement: Supplement 1 [file NIHPP2024.03.02.583119v1-supplement-1.pdf]

bioRxiv preprint doi: https://doi.org/10.1101/2024.03.02.583119; this version posted March 6, 2024. The copyright holder for this preprint (which was not certified by peer review) is the author/funder. This article is a US Government work. It is not subject to copyright under 17 USC 105 and is also made available for use under a CC0 license.

SUPPLEMENTAL FIGURES AND LEGENDS

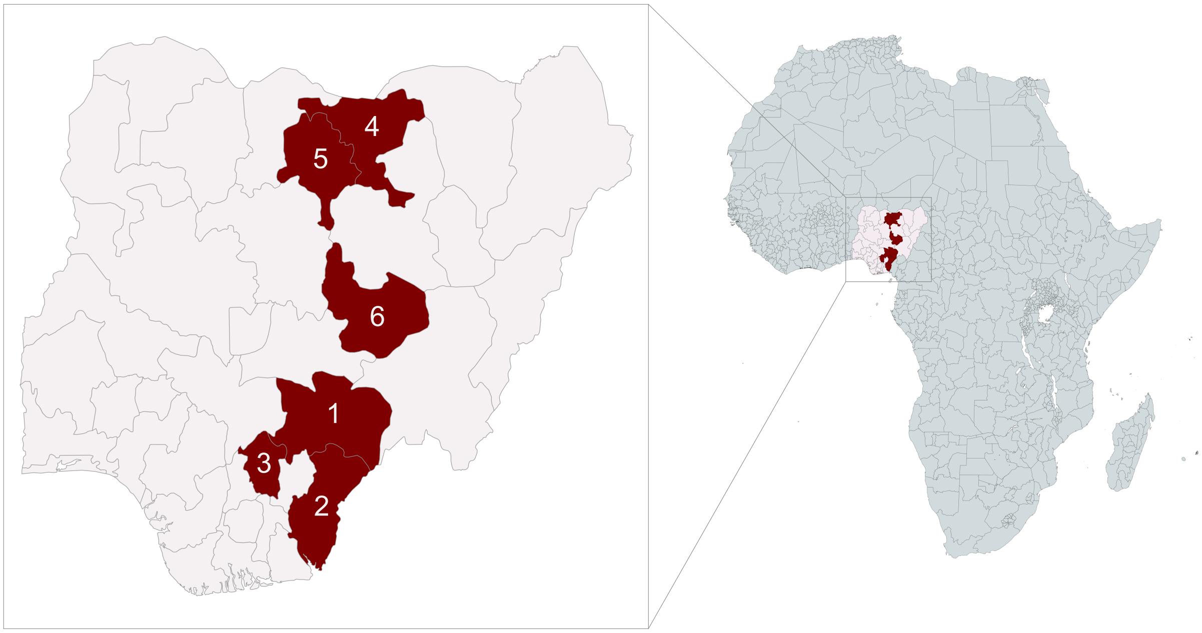

Supplemental figure 1: Nigeria map showing sampled states. 1. Benue; 2. Cross River; 3. Enugu; 4. Jigawa; 5. Kano; 6. Plateau.

bioRxiv preprint doi: https://doi.org/10.1101/2024.03.02.583119; this version posted March 6, 2024. The copyright holder for this preprint (which was not certified by peer review) is the author/funder. This article is a US Government work. It is not subject to copyright under 17 USC 105 and is also made available for use under a CC0 license.

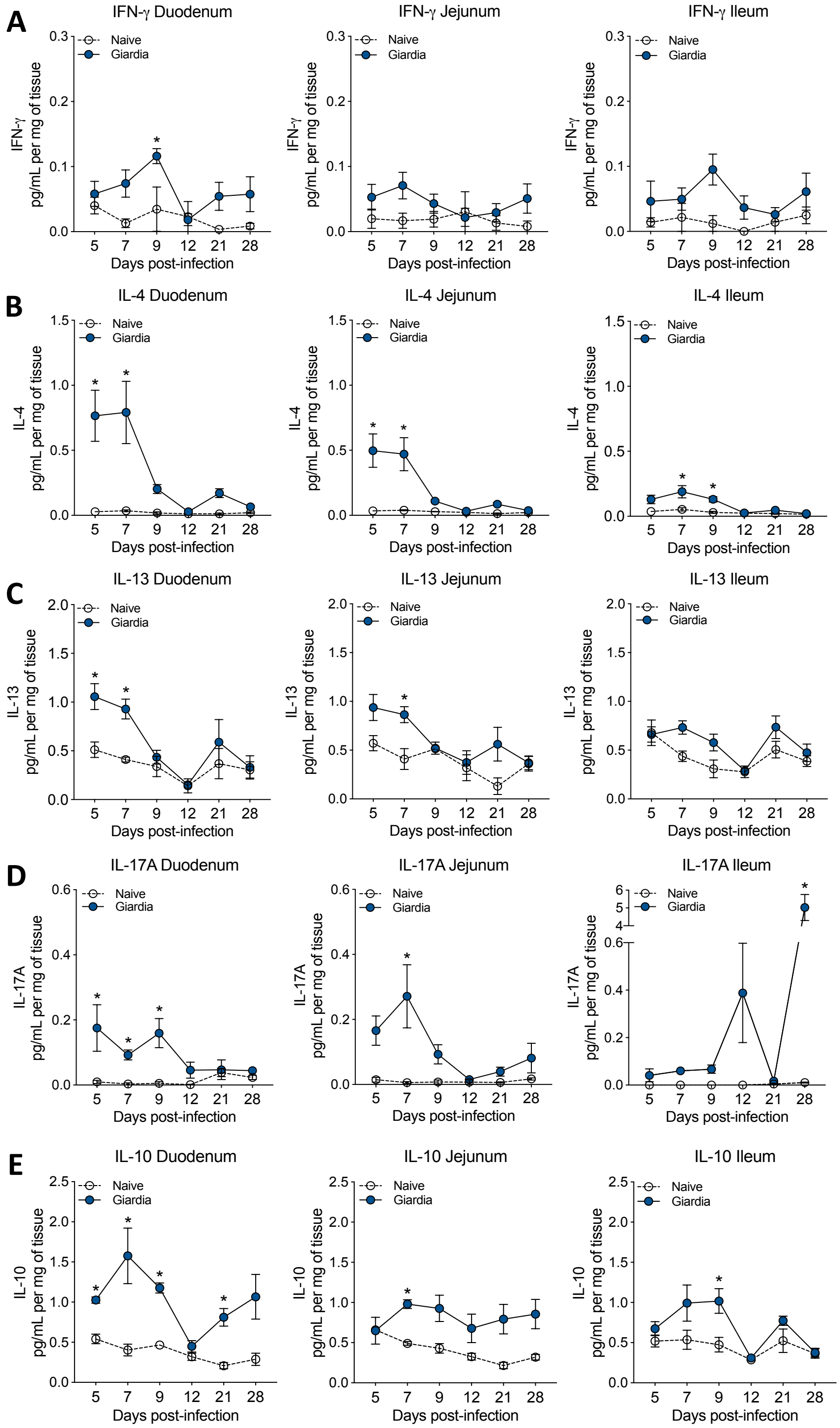

Supplemental figure 2: Cytokine production kinetics across the three segments from the small intestine in *Giardia*-infected mice. Levels of IFN-γ (A), IL-4 (B), IL-13 (C), IL-17A (D), and IL-10 (E) in the three different sections of the small intestine tissue homogenate (normalized by mg of tissue) from naïve or *Giardia*-infected mice at 5-, 7-, 9-, 12-, 21-, and 28-days post-infection (measured by Luminex). Data are represented as mean ± SEM for each time point and significance was calculated with one-way ANOVA test followed by Sidak's multiple comparisons test. *p≤0.05, **p≤0.01, ***p≤0.001. Data are representative of two independent experiments.

bioRxiv preprint doi: https://doi.org/10.1101/2024.03.02.583119; this version posted March 6, 2024. The copyright holder for this preprint (which was not certified by peer review) is the author/funder. This article is a US Government work. It is not subject to copyright under 17 USC 105 and is also made available for use under a CC0 license.

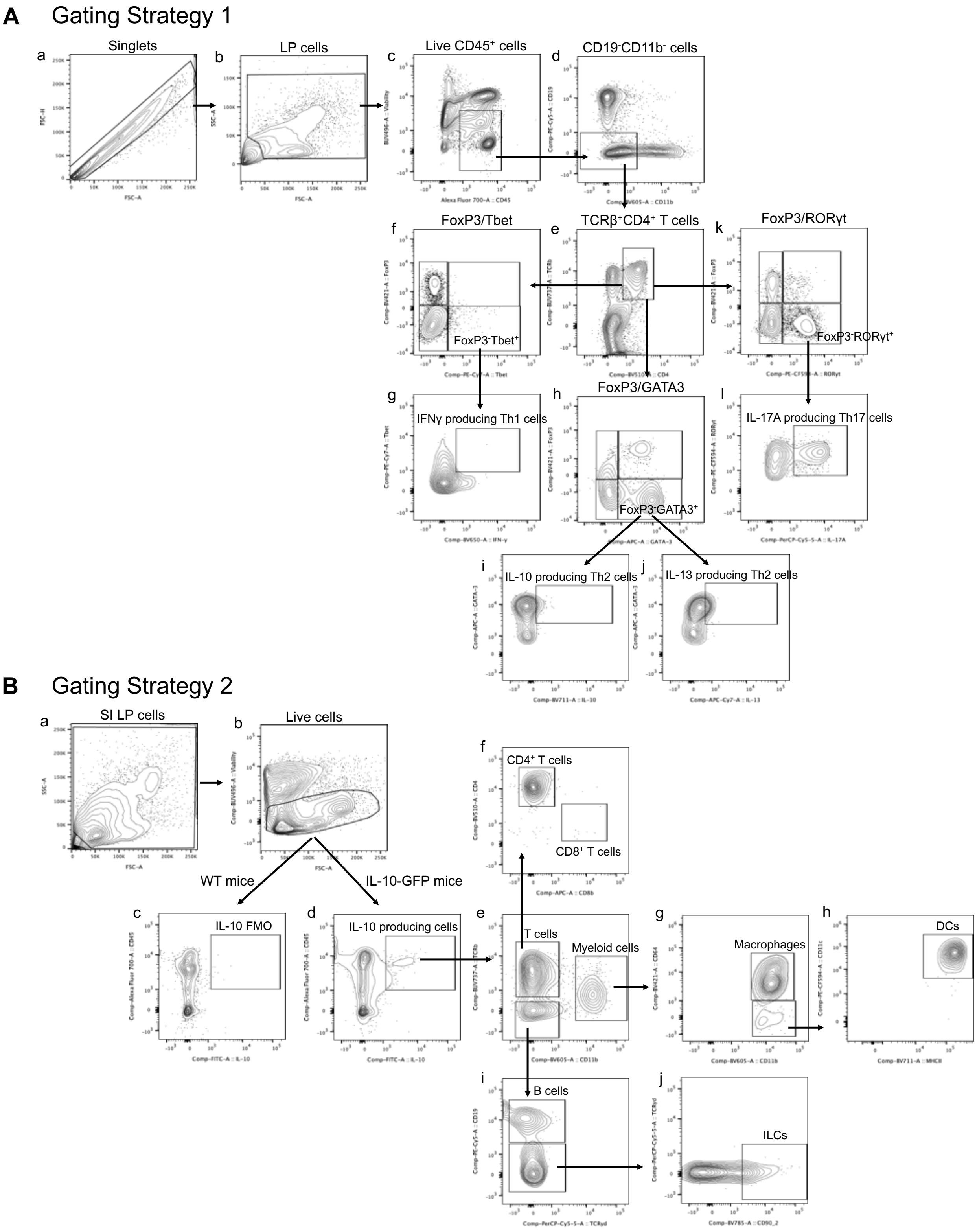

Supplemental figure 3: Gating strategy for immunophenotypic analysis by Flow Cytometry. (A) The gating strategy 1 was used for the overall immunophenotypic analysis of the experiments reported in figures 1B, 1G, 2D, 2G, 4B, 4C, 5E, 5J, 6E, and 6J. Briefly, singlets (a) were gated followed by subsequent lamina propria cell gating by FSC-A vs SSC-A (b). (c) Live hematopoietic cells were gated as CD45/Alexa Fluor700^{+} and Live/Dead/UV496^{-}. (d) B cells

bioRxiv preprint doi: https://doi.org/10.1101/2024.03.02.583119; this version posted March 6, 2024. The copyright holder for this preprint (which was not certified by peer review) is the author/funder. This article is a US Government work. It is not subject to copyright under 17 USC 105 and is also made available for use under a CC0 license.

and myeloid cells were then excluded by gating as CD19/PE-Cy5$^-$ and CD11b/BV605$^-$. (e) CD4$^+$ T cells were further gated as TCRβ/BUV737$^+$ and CD4/BV510$^+$. Subsets of CD4$^+$ T cells were further subdivided into either the single or co-expression of FoxP3/BV421 in combination with single or co-expression of Tbet/PE-Cy7 (f), GATA3/APC (h) or RORγt/PE-CF594 (k). Finally, IFN-γ/BV650 (g), IL-10/BV711 (i), IL-13/PE (j), and IL-17A/PerCP-Cy5.5 (l) were analyzed into the populations of Tbet$^+$ Th1 cells, GATA3$^+$ Th2 cells, and RORγt$^+$ Th17 cells, respectively. **(B)** The gating strategy 2 was design specifically for the identification of the major sources of IL-10 in the lamina propria of either naïve- and *Giardia*-infected IL-10 GFP reporter mice (Figure 2F). For this, live CD45/Alex Fluor 700$^+$ lamina propria cells (a, b) were gated for the IL-10 GFP expression (d), using a WT mouse as a control for a negative gate (c). (e) IL-10 producing hematopoietic cells were further characterized as either TCRβ/BUV737$^+$ T cells, or CD11b/BV605$^+$ cells or TCRβ$^-$CD11b$^-$ cells. (f) T cells were subdivided as CD4/BV510$^+$ cells or CD8β/APC$^+$ cells. (g) CD64/BV421$^+$ macrophages were identified within the CD11b$^+$ cells; and (h) CD64/BV421$^-$, CD11c$^+$/PE-CF596+, MHCII$^+$/BV711$^+$ cells were classified as Dendritic Cells (DCs). (i) Non-T and non-myeloid cells were further gated as CD19/PE-Cy5$^+$ cells or TCRγδ/PerCP.Cy5.5 T cells. (j) Finally, IL-10 producing CD45$^+$TCRβ$^-$CD11b$^-$CD19$^-$TCRγδ$^-$ CD90/BV785$^+$ cells were classified as innate lymphoid cells (ILCs).

bioRxiv preprint doi: https://doi.org/10.1101/2024.03.02.583119; this version posted March 6, 2024. The copyright holder for this preprint (which was not certified by peer review) is the author/funder. This article is a US Government work. It is not subject to copyright under 17 USC 105 and is also made available for use under a CC0 license.

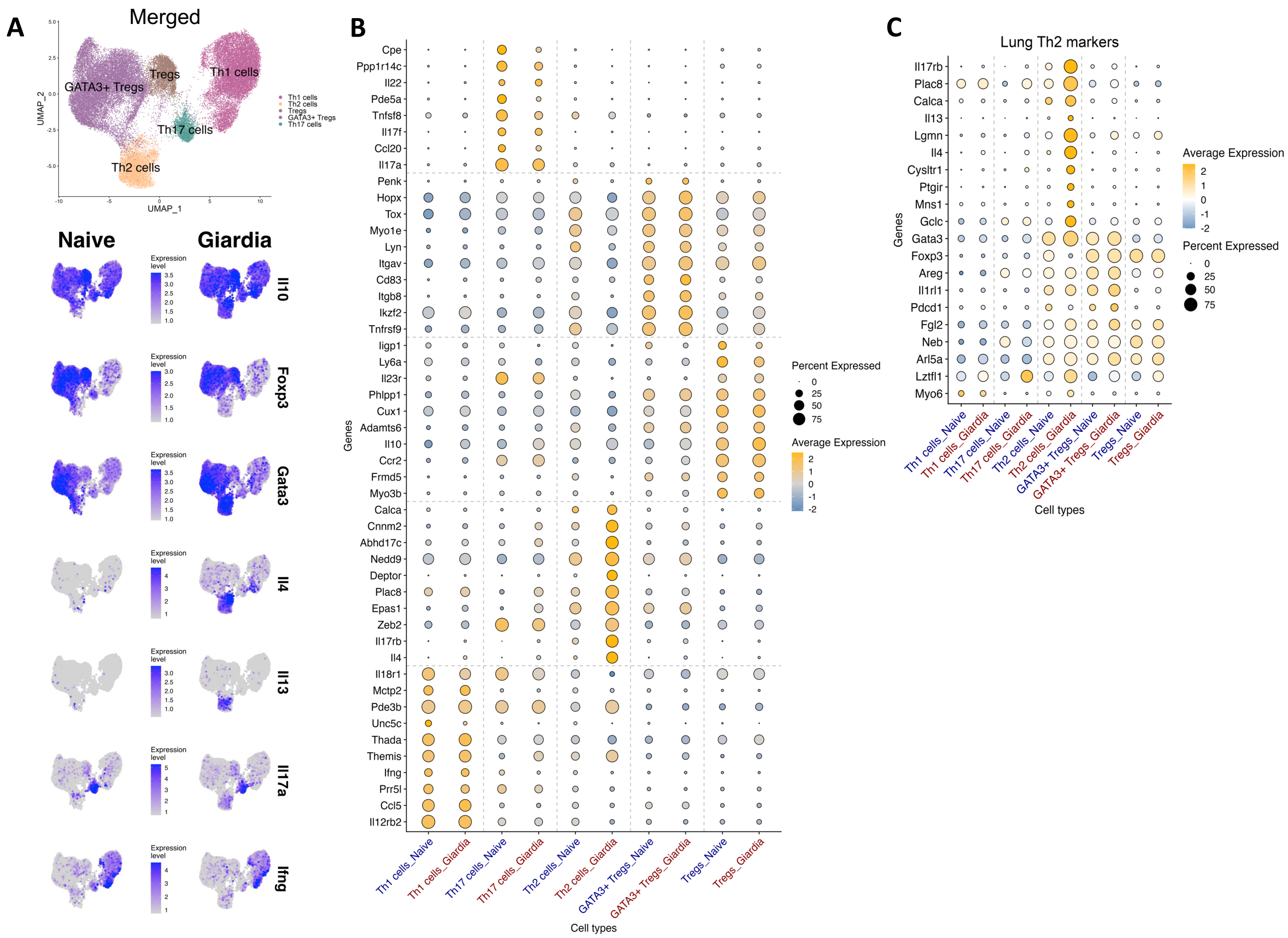

Supplemental figure 4: Molecular characterization of IL-10 producing CD4$^+$ T cells in the small intestine lamina propria. (A) UMAP plot showing the clustering analysis of the merged dataset of all sorted IL-10 producing CD4$^+$ T subsets in the small intestine lamina propria from both naïve and *Giardia*-infected IL-10-GFP reporter mice. The plot distinctly identifies five key clusters, corresponding to Th1, Th2, Th17, Treg, and Th2 Treg cells subset; and feature plot showing the gene expression level of *Il10*, *Foxp3* and *Gata3, Il4, Il13, Il17a, Ifng* among the major clusters of each IL-10 producing CD4$^+$ T subset from naïve (left) or *Giardia*-infected mice (right). (B) Dot plot graph highlighting the top10 highly expressed genes within each cluster of IL-10 producing CD4$^+$ T cells in the small intestine lamina propria from naïve and *Giardia*-infected mice. (C) Dot plot graph highlighting the signature genes of pathogenic Th2 cells within each cluster of IL-10 producing CD4$^+$ T cells in the small intestine lamina propria from naïve and *Giardia*-infected mice.

bioRxiv preprint doi: https://doi.org/10.1101/2024.03.02.583119; this version posted March 6, 2024. The copyright holder for this preprint (which was not certified by peer review) is the author/funder. This article is a US Government work. It is not subject to copyright under 17 USC 105 and is also made available for use under a CC0 license.

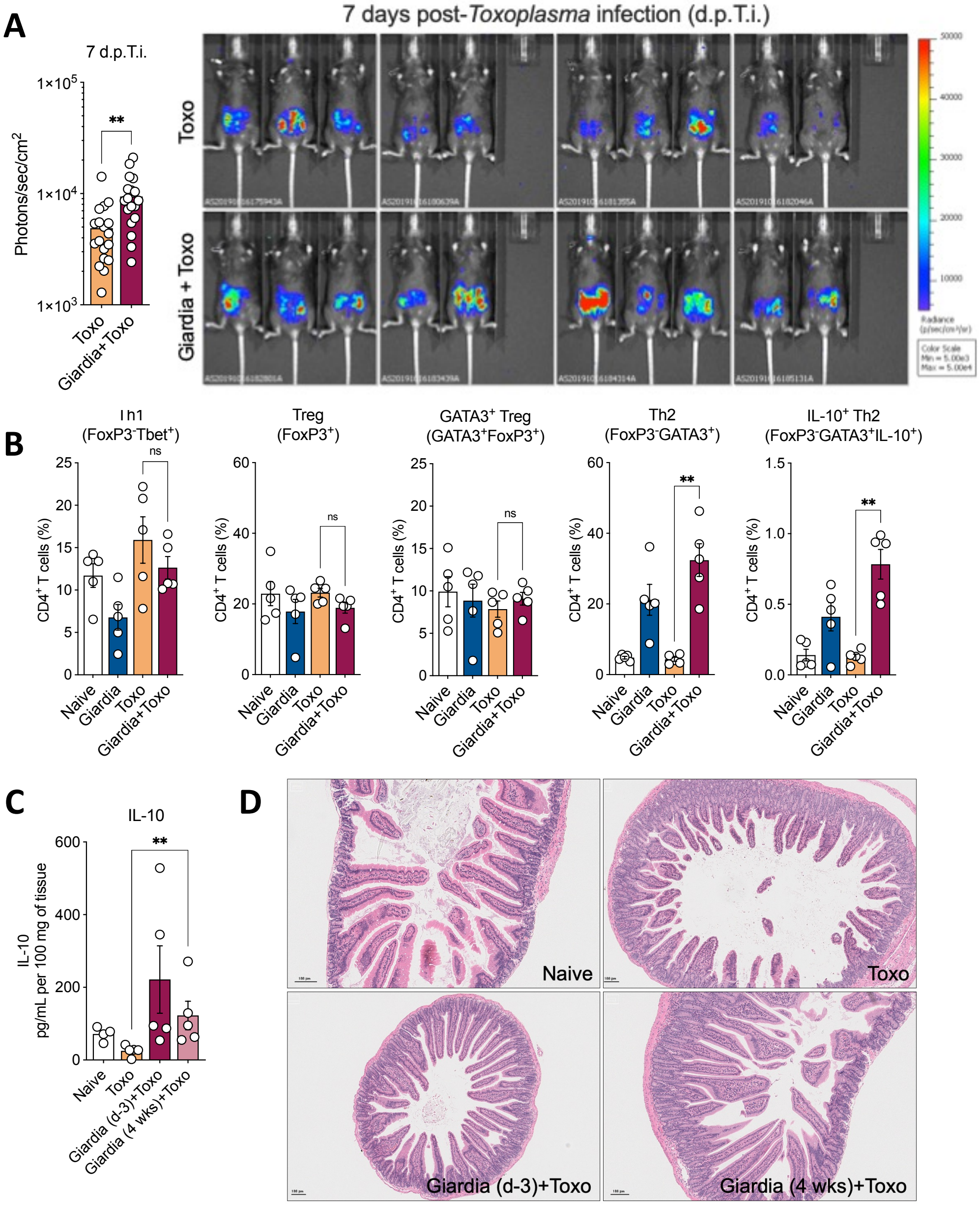

Supplemental figure 5: The effect of *Giardia* chronicity in the co-infection with *T. gondii*. (A) Bioluminescent detection in photons/sec/cm^2 shows *Toxoplasma* burden *in vivo* (7 d.p.T.i.) in mice co-infected or not with *Giardia* GS/M strain three days before. (B) Scatter plot graphs indicating the frequency of Th1 (FoxP3$^-$Tbet$^+$), total Treg (FoxP3$^+$), GATA3$^+$ Treg (GATA3$^+$FoxP3$^+$), Th2 (GATA3$^-$Foxp3$^+$), and IL-10$^+$ Th2 (FoxP3$^-$GATA3$^+$IL-10$^+$) cells in the small intestine lamina propria of *Giardia*- (three days before, d-3), *Toxoplasma*-, or *Giardia* (d-3)+*Toxoplasma*-infected mice 4 days post-*Toxoplasma* infection (4 d.p.T.i.). Gated on Live CD45$^+$TCRβ$^+$CD4$^+$. (C) IL-10 levels in the proximal small intestine of *Giardia*-chronically infected mice (4 weeks post-infection, 4 wks). (D) Representative image of H&E staining of the proximal small intestine from naïve, *Toxoplasma*-, *Giardia* (d-3)+*Toxoplasma*-, or *Giardia* (4 wks)+*Toxoplasma*-infected mice (8 days-post *Toxoplasma* infection). Scale bars represent 100 µm. Data are represented as mean ± SEM for each time point and significance was calculated with one-way ANOVA test followed by Sidak's multiple comparisons test. *p≤0.05, **p≤0.01, ***p≤0.001. Data are representative of two independent (A-B) and one (C-D) experiment.
